# AHR and NRF2 in Skin Homeostasis and Atopic Dermatitis

**DOI:** 10.3390/antiox11020227

**Published:** 2022-01-25

**Authors:** Tomohiro Edamitsu, Keiko Taguchi, Ryuhei Okuyama, Masayuki Yamamoto

**Affiliations:** 1 Department of Medical Biochemistry, Graduate School of Medicine, Tohoku University, Sendai 980-8575, Japan; etomohiro@shinshu-u.ac.jp (T.E.); keiko.taguchi.c8@tohoku.ac.jp (K.T.); 2 Department of Dermatology, Shinshu University Graduate School of Medicine, 3-1-1 Asahi, Matsumoto 390-8621, Japan; rokuyama@shinshu-u.ac.jp; 3 Department of Medical Biochemistry, Tohoku Medical Megabank Organization, Tohoku University, Sendai 980-8573, Japan; 4 Advanced Research Center for Innovations in Next-Generation Medicine (INGEM), Tohoku University, Sendai 980-8573, Japan

**Keywords:** AHR, NRF2, atopic dermatitis, skin, detoxification, keratinocyte differentiation, air pollutants, SNPs

## Abstract

Skin is constantly exposed to environmental insults, including toxic chemicals and oxidative stress. These insults often provoke perturbation of epidermal homeostasis and lead to characteristic skin diseases. AHR (aryl hydrocarbon receptor) and NRF2 (nuclear factor erythroid 2-related factor 2) are transcription factors that induce a battery of cytoprotective genes encoding detoxication and antioxidant enzymes in response to environmental insults. In addition to their basic functions as key regulators of xenobiotic and oxidant detoxification, recent investigations revealed that AHR and NRF2 also play critical roles in the maintenance of skin homeostasis. In fact, specific disruption of AHR function in the skin has been found to be associated with the pathogenesis of various skin diseases, most prevalently atopic dermatitis (AD). In this review, current knowledge on the roles that AHR and NRF2 play in epidermal homeostasis was summarized. Functional annotations of genetic variants, both regulatory and nonsynonymous SNPs, identified in the AHR and NRF2 loci in the human genome were also summarized. Finally, the possibility that AHR and NRF2 serve as therapeutic targets of AD was assessed.

## 1. Roles of AHR and NRF2 in Xenobiotic Detoxification

**Figure 1:**
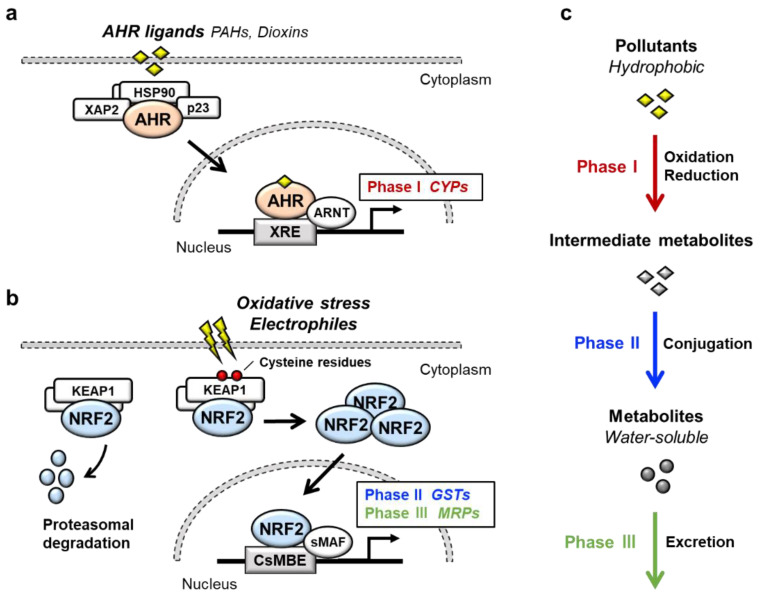
AHR and NRF2 in xenobiotic detoxification. (**a**) The AHR signaling pathway. In the absence of a ligand, AHR forms a complex with HSP90, p23 and XAP2 in the cytoplasm. Upon binding of ligands such as PAHs or dioxins, AHR translocates into the nucleus and forms a heterodimer with ARNT. The AHR-ARNT heterodimer binds to XRE in regulatory regions of target genes, which mainly encode phase I detoxification enzymes. (**b**) The KEAP1-NRF2 system. Under physiological conditions, KEAP1 binds and ubiquitinates NRF2 in the cytoplasm and promotes the degradation of NRF2 through the ubiquitin–proteasome pathway. Upon exposure to oxidative or electrophilic stresses, reactive cysteine residues of KEAP1 are modified, which leads to dysfunction of the ubiquitin ligase activity of KEAP1. Under this condition, newly made NRF2 escapes from KEAP1, translocates into the nucleus and forms a heterodimer with sMAF. The NRF2-sMAF heterodimer binds to CsMBE (also known as ARE or EpRE) in the regulatory regions of target genes, which mainly encode phase II detoxication enzymes and phase III transporters. (**c**) Xenobiotic detoxification. Xenobiotic detoxification consists of three phases. The phase I detoxication reaction involves oxidation or reduction of hydrophobic xenobiotics. The phase II detoxification reaction involves conjugation of the hydrophilic moiety by transferases to phase I metabolites, leading to the transformation of hydrophobic xenobiotics into water-soluble molecules. In the phase III reaction, the conjugated metabolites are eliminated from the cells through MRPs.

Transcription factor AHR (aryl hydrocarbon receptor) is a ligand-activated transcription factor that belongs to a basic helix-loop-helix/PER-ARNT-SIM (bHLH-PAS) family [1]. AHR is activated by the binding of a large variety of exogenous ligands, which include environmental pollutants such as polycyclic aromatic hydrocarbons (PAHs) and dioxins (Figure 1a). In the absence of a ligand, AHR forms a complex in the cytoplasm with at least three factors: Heat shock protein 90 (HSP90), the cochaperone protein p23 and hepatitis B virus X-associated protein 2 (XAP2) [2]. Upon binding of certain ligands to AHR, the AHR-HSP90-p23-XAP2 complex is disrupted, leading to the nuclear translocation of AHR. In the nucleus, AHR dimerizes with ARNT (AHR nuclear translocator) [3]. The AHR-ARNT heterodimer binds to XRE (xenobiotic response element) in regulatory regions of various AHR-target genes [4]. Prototype AHR-target genes include phase I detoxification enzymes such as cytochrome P450 (CYPs). Two AHR regulatory mechanisms have been reported. One is proteasomal degradation of the AHR protein, while the other is negative feedback regulation of AHR activation by the AHR repressor (AHRR) [5]. AHRR is known to be an AHR-target gene, and AHRR inhibits AHR activity by competing with AHR for dimerization with ARNT.

NRF2 (nuclear factor erythroid 2-related factor 2) is a transcription factor belonging to the cap‘n’collar (CNC) family. NRF2 possesses a basic leucine zipper (bZIP) and a CNC structure. NRF2 induces a battery of cytoprotective genes in response to oxidative and electrophilic stresses [6,7]. Under physiological conditions, NRF2 is bound by the repressor protein KEAP1 (kelch-like ECH-associated protein 1) in the cytoplasm, which promotes NRF2 degradation by the ubiquitin–proteasome pathway (Figure 1b) [8,9]. Upon exposure to oxidative or electrophilic stresses, specific reactive cysteine residues of KEAP1 are modified, which leads to the disruption of the KEAP1-NRF2 interaction [10,11]. This stabilizes NRF2 so that NRF2 translocates to the nucleus and forms a heterodimer with small MAF (sMAF) protein. The NRF2-sMAF heterodimer complex binds to the CNC-sMAF binding element (CsMBE), which is also known as the antioxidant response element (ARE) or electrophile response element (EpRE), in the regulatory regions of target genes [12,13]. Closer examination of the NRF2-target genes revealed that a set of genes encoding phase II detoxification enzymes such as glutathione *S*-transferase (GSTs), antioxidative enzymes such as NAD(P)H:quinone oxidoreductase 1 (NQO1), glutamate-cysteine ligase catalytic subunit (GCLC) and phase III transporters such as multidrug-resistance-associated proteins (MRPs) in a member of ATP-binding cassette (ABC) transporters are the target genes of NRF2.

AHR and NRF2 are the key regulators of cytoprotective responses to environmental stresses. Both transcription factors play important roles in the transformation of hydrophobic molecules to water-soluble molecules that are easily eliminated from the body via urine, stool and sweat. Xenobiotic detoxification consists of three phases [14] (Figure 1c); the phase I detoxification reaction involves oxidation or reduction of xenobiotics, resulting in the conversion to more polar intermediate metabolites such as electrophiles. The phase II detoxification reaction involves conjugation of a hydrophilic moiety, e.g., glucuronate, sulfate, glutathione or glycine, to phase I metabolites. This reaction is catalyzed by a group of enzymes called transferases, and phase I metabolites are transformed into water-soluble molecules by transferases. In the phase III reaction, the conjugated metabolites are eliminated from the cells by transporters. It has been shown that AHR regulates the expression of phase I enzymes, such as CYP1A1, CYP1A2 and CYP1B1, while NRF2 regulates phase II enzymes, such as GSTA1, GSTP1 and UDP-glucuronosyl transferases (UGTs), and phase III transporters, such as MRPs.

## 2. Crosstalk between AHR and NRF2

**Figure 2:**
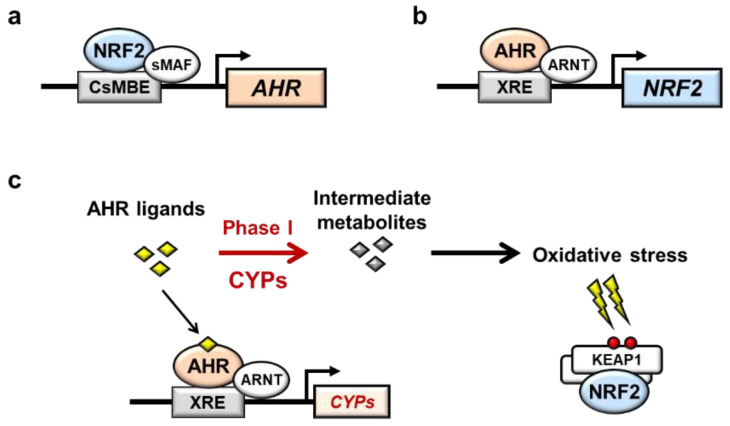
Interaction between AHR and NRF2. (**a**) Direct interaction between AHR and NRF2. NRF2 directly binds to a CsMBE in the *AHR* promoter and increases the transcription of *AHR* mRNA. (**b**) In contrast, AHR directly binds to an XRE in the *NRF2* promoter and increases the transcription of *NRF2* mRNA. (**c**) Indirect interaction between AHR and NRF2 through AHR-mediated oxidative stress. AHR ligands are metabolized by AHR-induced CYPs. Intermediate metabolites often induce oxidative stress, resulting in activation of NRF2.

The relationship between AHR and NRF2 has attracted attention, and a reciprocal relationship between AHR and NRF2 has been reported. First, Shin et al. showed that pharmacological activation of Nrf2 by CDDO-Im, 1-(2-cyano-3,12,28-trioxooleana-1,9(11)-dien-28-yl)-1H-imidazole, induces *AhR* mRNA and the mRNA expression of the target genes *Cyp1a1* and *Cyp1b1* in mouse embryonic fibroblasts (MEFs), indicating that Nrf2 directly regulates the transcription of *AhR* mRNA (Figure 2a) [15]. The induction of the *AhR*, *Cyp1a1* and *Cyp1b1* genes was cancelled in *Nrf2*-knockout MEFs, and luciferase assays and chromatin immunoprecipitation (ChIP) assays showed that Nrf2 directly binds to a CsMBE at −230 of the *AhR* promoter [15].

In contrast, 2,3,7,8-tetrachlorodibenzo-*p*-dioxin (TCDD), a high affinity AhR ligand, was found to induce *Nrf2* mRNA expression in an AhR-dependent manner in mouse hepatoma 1c1c7 cells, suggesting that AhR directly increased the transcription of *Nrf2* mRNA (Figure 2b) [16]. Direct binding of AhR to the XRE-like elements at −712, +755 and +850 of the *Nrf2* gene was confirmed by ChIP assay, and luciferase assay further revealed that TCDD induces *Nrf2* gene expression through all three XRE-like elements [16]. In this regard, while it has been shown that NRF2 activation heavily depends on the stabilization of NRF2 protein from proteasome degradation [8,9], there are reports that the transcriptional activation of NRF2 contributes to cellular NRF2 activity [17].

Therefore, it is interesting to note that in addition to the reports suggesting the direct relationship of AHR and NRF2, there are reports that suggest indirect interactions between AHR and NRF2 (Figure 2c). NRF2 is known to be activated by oxidative stresses, which are increased through the induction of CYPs. For instance, TCDD induces *NQO1* gene expression in human hepatoma cell lines, but antioxidant *N*-acetyl cysteine (NAC) or *CYP1A1* siRNA decreases *NQO1* gene induction, indicating that *NQO1* induction is controlled by *CYP1A1* activity through oxidative stress [18]. TCDD activates Nrf2 activity in vivo in an AhR-dependent manner, leading to the induction of Nrf2 target genes, including *Nqo1* and *Gsta1*, in mouse livers [19]. In contrast, Nrf2 target gene expression induced by TCDD could not be cancelled in the liver of *Cyp1a1*/*1a2*/*1b1* triple-knockout mice, suggesting that AhR itself induces Nrf2 independent of Cyp enzyme activity [20]. Therefore, it appeared that AHR ligands directly or indirectly influence NRF2 activity, but further studies are needed to clarify the relationships between AHR activation and NRF2 induction.

## 3. Barrier Function of the Skin

**Figure 3:**
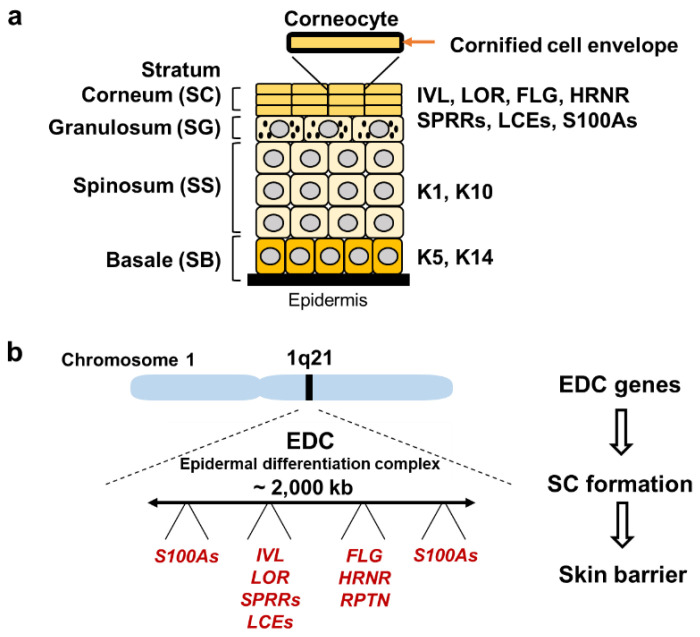
Structure of the epidermis and genes regulated by AHR and NRF2. (**a**) Structure of the epidermis. The epidermis includes four layers: SC, SG, SS and SB. Through cell migration from SB toward SC, keratinocytes start differentiation and sequentially express specific proteins, such as Keratin 5/14 at the SB stage and Keratin 1/10 at the SS stage. The outer layer SC consists of corneocytes, which are surrounded by a cornified cell envelope. SC expresses IVL, LOR, FLG, SPRRs, LCEs and the S100A family. (**b**) EDC on human chromosome 1q21. EDC genes encode the components of SC and function in the formation of the skin barrier.

The primary function of the skin is a surface barrier, which prevents dehydration and protects the body from environmental insults. Therefore, impairment of skin barrier function facilitates the absorption of environmental toxicants and leads to enhanced water loss. The skin consists of two layers, epidermis and dermis, and the epidermis includes four cell layers that mainly consist of keratinocytes (Figure 3a): The stratum corneum (SC), stratum granulosum (SG), stratum spinosum (SS) and stratum basale (SB).

Barrier function of the skin mainly depends on SC, the outermost layer. SC consists of terminally differentiated keratinocytes, named corneocytes, which express involucrin (IVL), loricrin (LOR), filaggrin (FLG), small proline-rich proteins (SPRRs), late cornified envelope proteins (LCEs) and the S100A family. IVL, LOR, SPRRs and LCEs are the major components of the cornified cell envelope, a highly insoluble structure inside the plasma membrane of corneocytes. FLG is a complex mixture of water-soluble compounds such as amino acids, and is one of the major compounds of natural moisturizing factors in the epidermis. Most of the genes encoding the components of the cornified cell envelope are clustered in the epidermal differentiation complex (EDC) on human chromosome 1q21 (Figure 3b).

## 4. Role of AHR in Epidermal Homeostasis

A well-known function of AHR is the regulation of xenobiotic metabolism through the induction of CYPs. However, many lines of evidence have reported that ligand-activated AHR also acts to induce the expression of genes involved in skin barrier formation [21,22,23,24]. In fact, exposure of normal human epidermal keratinocytes to TCDD increases the mRNA expression of 40% (24/60) of the EDC genes involved in cornified cell envelope formation, such as *FLG*, *FLG2*, *LCE1C*, *LCE2A*, *LCE2B*, *LCE3A*, *LCE3E*, *SPRR1A*, *SPRR2A*, *SPRR2B*, *S100A9*, *S100A12*, *S100A7*, *Repetin* (*RPTN*) and *Hornerin* (*HRNR*) [23,24]. In organotypic cultures of human keratinocytes, treatment with TCDD causes well-developed SC, indicating that TCDD accelerates the onset of epidermal terminal differentiation [21]. Additionally, exposure to TCDD accelerates the formation of the fetal mouse skin barrier in utero [23]. Conversely, in primary keratinocytes derived from wild-type mice, the expression of terminal differentiation genes is suppressed by treatment with an AhR antagonist GNF351 or a selective AhR modulator SGA360 [25]. The expression of terminal differentiation genes is reproducibly repressed in AhR-knockout mouse primary keratinocytes [25]. These results support the physiological roles of AhR during epidermal differentiation.

AhR-knockout mice were generated in 1996–1997 independently in three distinct laboratories (Table 1) [26,27,28]. Fernandez-Salguero et al. reported an AhR-knockout mouse line generated by replacing a part of the first exon of the *AhR* gene with a neomycin resistance cassette (NEO). The mice revealed severe hyperkeratosis, acanthosis and marked dermal fibrosis in the dorsal skin [26]. In contrast, the other two lines of AhR-knockout mice did not show characteristic skin phenotypes. The second and third knockout mouse lines were generated by the replacement of a part of the second exon with NEO and LacZ fused to a nuclear localization sequence, respectively [27,28]. While these differences in gene targeting strategy may contribute to the phenotypic difference of the skin, the precise reason for the phenotypic difference is currently unclear.

Consistent with the results of the latter two mouse lines, keratinocyte-specific AhR-knockout mice using Keratin 14 (K14)-Cre (AhR^flox^::K14-Cre mice) show no obvious macroscopic phenotypes in unstressed skin [29]. However, when the upper layers of SC are mechanically removed by tape stripping, transepidermal water loss (TEWL) values in AhR^flox^::K14-Cre mice are substantially increased compared with those in control mice, suggesting that AhR plays a role in skin barrier function [29]. These studies using AhR-knockout cell lines and AhR-knockout mice support the notion that AHR is involved in epidermal homeostasis [25,26], although there remain many unsolved questions.

ARNT is indispensable for the transcriptional activity of AHR. Keratinocyte-specific Arnt-knockout mice using Keratin 5 (K5)-Cre (Arnt ^flox^::K5-Cre mice) show severe impairment of the epidermal barrier [30]. Arnt ^flox^::K5-Cre mice exhibit a loss of body weight and die within 24 h after birth [30]. TEWL is increased in the epidermis of Arnt ^flox^::K5-Cre mice, and the application of salve to the skin retards weight loss; the mice survive longer than 24 h [30]. These observations indicate that skin barrier dysfunction results in severe dehydration in Arnt ^flox^::K5-Cre mice.

**Table 1:** Genetically modified mouse models targeting AhR/Arnt and Keap1/Nrf2.

Mouse	Reference			Main Skin Phenotype
**AhR**				
AhR-knockout	Schmidt et al.	1996	[28]	No obvious phenotypes in normal condition
	Mimura et al.	1997	[27]	
	Fernandez-Salguero et al.	1997	[26]	Hyperkeratosis, acanthosis and dermal fibrosis
AhR^flox^::K14-Cre	Haas et al.	2016	[29]	Increased TEWL after tape stripping
AhR-CA	Tauchi et al.	2005	[31]	Hyperkeratosis, acanthosis and dermal infiltration with inflammatory cellsFrequent scratching behavior
			[32]	
**Arnt**				
Arnt^flox^::K14-Cre	Takagi et al.	2003	[30]	Increased TEWL and postnatal death
Arnt^flox^::K5-Cre	Geng et al.	2006	[33]	
**Keap1**				
Keap1-knockout	Wakabayashi et al.	2003	[34]	Hyperkeratosis
				Death before weaning age due to obstruction of the esophagus and forestomach
**Nrf2**				
caNrf2::K5-Cre	Schäfer et al.	2012	[35]	Hyperkeratosis, acanthosis, cyst formation andenlarged sebaceous glands
		2014	[36]	
*Lor*-knockout::dnNrf2	Huebner et al.	2012	[37]	Failure in skin barrier formation and postnatal death

Consistent with the results of the Arnt ^flox^::K5-Cre mice, keratinocyte-specific *Arnt*-knockout mice using K14-Cre (Arnt ^flox^::K14-Cre mice) also die with a failure of epidermal barrier function [33]. Microarray analyses of the epidermis of Arnt ^flox^::K14-Cre mice revealed upregulation of several EDC genes, including *S100a* genes (*S100a8*, *S100a9*, *S100a10*) and *Sprrs* (*Sprr1a*, *Sprr2i*, *Sprr2j*, *Sprrl1*) [33]. Furthermore, serine protease inhibitors, including secretory leukocyte protease inhibitor (*Slpi*), are also upregulated and are involved in SC desquamation [33]. While Hif1α (hypoxia-inducible factor 1α) can also form a heterodimer with Arnt, keratinocyte-specific Hif1α-knockout mice using K5-Cre (Hif1α^flox^::K5-Cre mice) show no obvious phenotype, suggesting that the AhR-Arnt heterodimer rather than Hif1α-Arnt is responsible for epidermal homeostasis [30]. These wide-ranging reports suggest that AhR or Arnt deficiency impairs the development and function of the epidermal barrier, but further clarifications are required to understand the general features.

## 5. Role of NRF2 in Epidermal Homeostasis

Nrf2 is involved in the regulation of epidermal homeostasis, and analyses of several genetically modified mice support this notion (Table 1). Systemic Keap1-knockout mice in which Nrf2 activity is constitutively activated macroscopically exhibit scaling skin, and histological examinations reveal the presence of severe hyperkeratosis in the skin, esophagus and forestomach [34]. Keap1-knockout mice die before weaning due to obstruction of the esophagus and forestomach caused by hyperkeratosis of the squamous cell epithelium [34]. As epithelial dysfunction can be rescued by concomitant *Nrf2* gene deletion, high-level activation of Nrf2 in keratinocytes caused by *Keap1* deletion must be the reason for epithelial abnormalities [34].

Showing very good agreement with the results of Keap1-knockout mice, a transgenic mouse line expressing a constitutively active Nrf2 mutant (caNrf2) specifically in keratinocytes was shown to display scaling and dry skin [35]. caNrf2 lacks the Neh2 domain, which is responsible for binding to Keap1, and this molecule is expressed under the control of a strong CMV enhancer and specific removal of the stopper cassette by K5-Cre (caNrf2::K5-Cre mouse). Histological analyses of the caNrf2 transgenic mice showed thickening of the epithelium (acanthosis) and severe hyperkeratosis in the skin. Microarray analyses using the skin of caNrf2::K5-Cre mice at postnatal Day 2.5 revealed upregulation of genes involved in epidermal barrier formation, including *Slpi*, *Sprr2d* and *Sprr2h* [35]. These mouse phenotypes suggest that prolonged activation of Nrf2 in keratinocytes disturbs skin homeostasis.

Supporting this notion, this research group has also reported that Nrf2 activation in newborn mice induced by topical application with sulforaphane or *tert*-butyl hydroquinone (tBHQ) for 10 days also causes skin abnormalities resembling those of caNrf2::K5-Cre mice [35]. However, the concentrations of the inducers applied were 10 mM and 50 mM for sulforaphane and tBHQ, respectively, which are very high doses. Therefore, the relationship between pharmacological induction of skin phenotypes and Nrf2 contribution awaits further verification.

In this regard, crossing a transgenic mouse line expressing the dominant-negative form of Nrf2 (dnNrf2) under the control of the *Lor* promoter to *Lor*-knockout mice (*Lor*-knockout::dnNrf2 mice) showed a distinct phenotype. Inhibition of endogenous Nrf2 activity by dnNrf2 in the *Lor*-deficient epidermis causes severe loss of barrier function and death within 24 h [37]. LOR is the main component of the cornified cell envelope. While *Lor*-knockout mice show a delay in the formation of the epidermal barrier in utero, the mice can survive with the formation of a functional barrier by birth [38]. Other cornified cell envelope components, including *Sprrs* and *Lce1*, are upregulated in the epidermis of *Lor*-knockout mice, so they might compensate for the loss of *Lor* [38,39]. In fact, the expression of *Sprrs* and *Lce1* genes is decreased to basal levels in the epidermis of *Lor*-knockout::dnNrf2 mice [37,39]. ChIP assays and reporter assays show that Nrf2 directly upregulates the *Sprrs* (*Sprr2d* and *Sprr2h*) and *Lce1 genes* (*Lce1b, Lce1c, Lce1e, Lce1g, Lce1h* and *Lce1m*) [37,39]. These results suggest that Nrf2 activation in *Lor*-knockout mice may act to induce a compensatory response to repair the defective barrier. While the Keap1-Nrf2 system is generally the major cellular protection mechanism against xenobiotic and oxidative stresses, these reports suggest that Nrf2 plays important roles in the maintenance of skin homeostasis, similar to AhR.

## 6. AHR as Therapeutic Target for Atopic Dermatitis

Atopic dermatitis (AD) is a chronic recurrent inflammatory skin disease characterized by epidermal barrier dysfunction and immune dysregulation. Loss-of-function variants in *FLG*, which is essential for the formation of the epidermal barrier, have been reported to be the most common risk factor for the development of AD [40]. However, only 40% of individuals with *FLG* variations develop AD symptoms [40,41,42]. These facts indicate that other factors are also involved in the pathogenesis of AD [42,43].

It has been shown that AD patients account for up to 25% of children and 2–3% of adults [44]. Of note, the international study of asthma and allergies in childhood (ISAAC), which is a worldwide epidemiological research in more than 50 countries, revealed that the prevalence of AD increased among schoolchildren between ISAAC phase I (1992–1996) and phase III (2000–2003) studies in many parts of the world [45]. In addition, a systematic review including 69 reports showed that the prevalence of AD between 1990 and 2010 increased in Africa, East Asia and parts of Europe [46]. While the pathophysiology of AD appears to be multifactorial with complex interactions between genetic and environmental factors, the recent increase in the prevalence of AD supports the attribution of environmental factors in predisposed individuals [47].

In fact, several lines of epidemiological research provide evidence that exposure of the skin to air pollutants acts as a risk factor for the development or aggravation of AD (Table 2). Outdoor air pollutants are mostly generated from the burning of fossil fuels for vehicles and industries [48], and components of air pollutants mainly include particulate matter (PM), which is a mixture of solid or liquid particles suspended in the air. Outdoor concentrations of PM are associated with increased AD symptoms and itching [49,50]. A meta-analysis of 13 studies showed positive correlations between PM exposure and AD [51]. Importantly, PM contains PAHs, prototypical AHR ligands, and induces *CYP1A1* mRNA expression in an AHR-dependent manner in human primary keratinocytes [52]. In addition to PM, active smoking and passive exposure to tobacco in the home are also associated with a higher prevalence of AD in both children and adults [53]. Tobacco smoke is the major source of indoor pollutants and contains numerous chemical constituents, including PAHs [54]. These reports support the hypothesis that AHR activation is involved in AD caused by air pollutants.

A model study employing constitutively active AhR in mice supported the hypothesis that AhR plays an important role in the development of AD [31]. To analyze the effects of chronic AhR activation in the epidermis, a transgenic mouse expressing a constitutively active form of AhR was generated under the control of the *K14* promoter (AhR-CA mice; Table 1). AhR-CA mice develop erosive eczema in the face and back skin with highly frequent scratching [31]. Histological examination shows acanthosis, hyperkeratosis and abundant infiltration of cells related to Th2-type inflammation in the skin [32]. These phenotypes highly recapitulate those of patients with AD, indicating that the constitutive activation of AhR provokes pathological conditions similar to those frequently observed in patients with AD.

Notably, in the epidermis of AhR-CA mice, the neurotrophic factor Artemin is highly expressed, which causes extension of sensory nerves into the epidermis and provokes hypersensitivity to itch or alloknesis [32,57]. The *Artemin* gene is directly regulated by AhR via an XRE-containing enhancer located 52 kb upstream of the gene [58]. Showing very good agreement with these results, in the skin of AD patients, AHR is activated, followed by upregulation of the mRNA and protein levels of CYP1A1 and ARTEMIN [32,59].

In contrast, it has been reported that AHR activation improves skin barrier function by accelerating epidermal terminal differentiation. For instance, coal tar has been used as a therapeutic agent for inflammatory skin diseases, including AD. In fact, topical application of coal tar restores the expression of skin barrier proteins, including FLG, in the skin of patients with AD [60]. Coal tar contains abundant PAHs and has been shown to induce epidermal differentiation via AHR. The efficacy of coal tar seems to be attributable to AHR activation [60].

**Figure 4:**
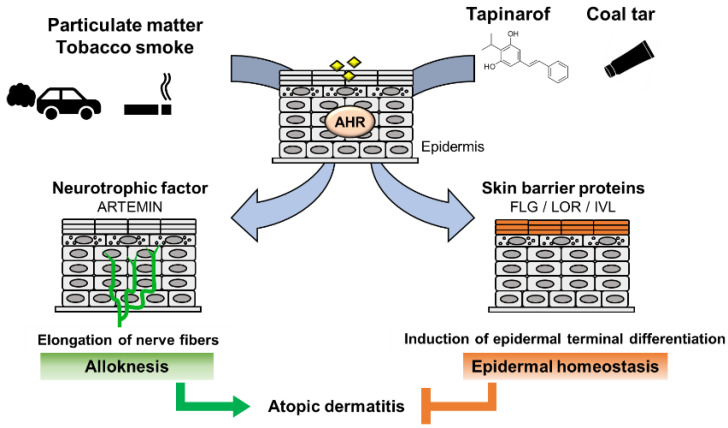
Dual functions of AHR in atopic dermatitis. PM and tobacco smoke contain PAHs that activate AHR. Activation of AHR induces the expression of the neurotrophic factor Artemin. Upregulation of Artemin causes extension of cutaneous sensory nerves into the epidermis and provokes hypersensitivity to itch or alloknesis. Tapinarof and coal tar improve skin barrier function by accelerating epidermal terminal differentiation.

In addition, tapinarof, 3,5-dihydroxy-4-isopropyl-*trans*-stilbene, a natural origin small molecule, shows efficacy in patients with AD and is now in clinical development for the treatment of skin diseases. In a phase 2 double-blind study, AD symptom was improved in the patients treated with tapinarof cream [61,62]. Recent research revealed that tapinarof activates AHR through direct binding as an agonist and induces mRNA expression of epidermal terminal differentiation markers, including *FLG* and *IVL*, in primary human keratinocytes [63]. Imiquimod has been used to provoke skin inflammation that mimics psoriasis, and tapinarof reduces imiquimod-induced inflammation in mouse skin in an AhR-dependent manner [63]. Similar to the case for coal tar, the efficacy of tapinarof is considered to depend, at least partially, on the AhR activity. These wide-ranging observations thus demonstrate that AHR activation acts to repair skin barrier function, but in turn, excessive expression of AHR leads to the development of AD (Figure 4). These reports demonstrated that AHR is a potential therapeutic target, and moderate-level activation of AhR may have therapeutic efficacy for skin barrier recovery. In contrast, AHR inhibitors seem to be important for the treatment of AD.

## 7. Genetic Factors Affecting the NRF2 Activities

**Figure 5:**
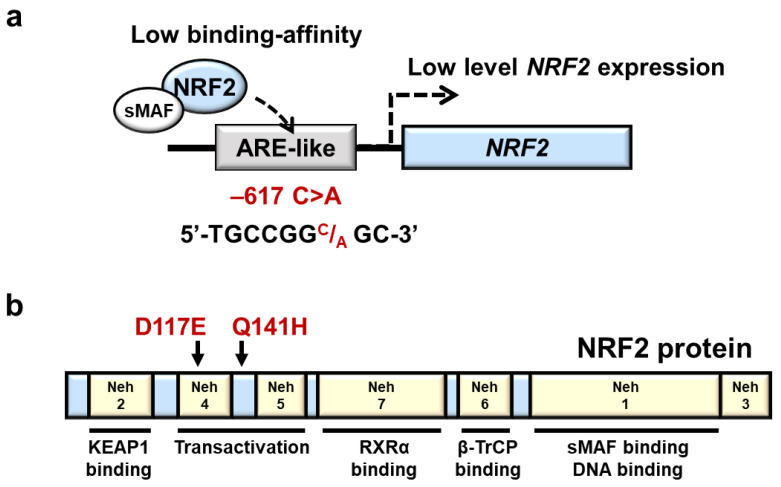
A regulatory SNP and two nonsynonymous SNPs in the *NRF2* gene. (**a**) Location of *NRF2* rSNP-617 in the ARE-like motif in the upstream promoter region of the *NRF2* gene. rSNP −617 C>A decreases the affinity of NRF2 binding to the ARE-like motif. (**b**) Location of nonsynonymous *NRF2* SNPs associated with NRF2 domain structures. Human NRF2 protein contains 7 Neh domains. Neh, Nrf2-ECH homology.

The human *NRF2* gene is located on chromosome 2q31.2, and according to the single nucleotide polymorphism database (dbSNP), there are multiple SNPs in the *NRF2* locus. One salient example is a regulatory SNP (rSNP) −617 C>A (rs6721961), which is in the ARE-like motif in the upstream promoter region of the *NRF2* gene (Figure 5a). The minor allele frequency (MAF) of *NRF2* −617 C>A varies among populations and is relatively high in Asians; the MAF is 0.07 in Africans, 0.21 in Asians and 0.12 in Europeans according to the dbSNP. In fact, the frequency is also high in the Japanese population, but it varies depending on the research: 0.25 in jMorp 14KJPN (Japanese multi-omics reference panel) and 0.28 in the Iwaki health promotion project [64,65].

It has been shown that the luciferase activity of a promoter construct with the −617 A/A variant is lower than that of a construct bearing the −617 C/C variant when transfected into A549 cells [66]. Electrophoretic mobility shift assay (EMSA) has shown that the formation of NRF2 protein-DNA complex in the ARE-like sequence is reduced in the C/A and A/A genotypes compared with the C/C genotypes, indicating that the rSNP −617 C>A decreases the affinity of NRF2 binding to the ARE-like motif, resulting reduced self-induction of NRF2 [66]. Supporting this notion, the minor A/A genotype at −617 exhibits a lower level of *NRF2* mRNA expression in human lymphocytes by approximately 40% than the C/C and C/A genotypes [17]. The expression levels of tBHQ-induced NRF2 protein and *NQO1* mRNA in A/A genotypes were lower than those in C/C genotype lymphocytes [17]. It has been shown that the rSNP at −617 C>A is associated with a higher risk of various oxidative stress-involved diseases, including acute lung injury [66], non-small-cell lung cancer in smokers [17], noise-induced hearing loss [67], venous thromboembolism [68] and vascular stiffness with aging [64].

In addition to the rSNP at −617 C>A, many nonsynonymous SNPs are found and reported in the coding region of the human *NRF2* gene [69,70,71]. Of the nonsynonymous SNPs, the following two are interesting to note (Figure 5b). D117E (351 T>A, rs1469602964) and Q141H (423 G>T, undetermined rs number) variants of NRF2 were found to enfeeble NRF2 activity in an experiment utilizing 293T culture cells [71]. Furthermore, the mRNA levels of NRF2 target genes, including *GSTP1*, *GSTM1* and heme oxygenase-1 (*HO-1*), are reduced in D117E- and Q141H-expressing 293T cells compared with cells expressing wild-type NRF2, and these NRF2 variants are proposed to be associated with the pathogenesis of Parkinson’s disease in Chinese populations [71]. These are intriguing observations suggesting the roles that NRF2 plays in neuroinflammatory diseases but need to be verified further in successive cohort studies.

While there is no report on the relationship of the *NRF2* SNPs with AD pathogenesis, the polymorphisms of *GSTM1* and *GSTT1*, which are members of the *GST* gene family and NRF2 target genes, are reported to increase the risk of AD [72].

## 8. Genetic Factors Affecting the AHR Activity

**Figure 6:**
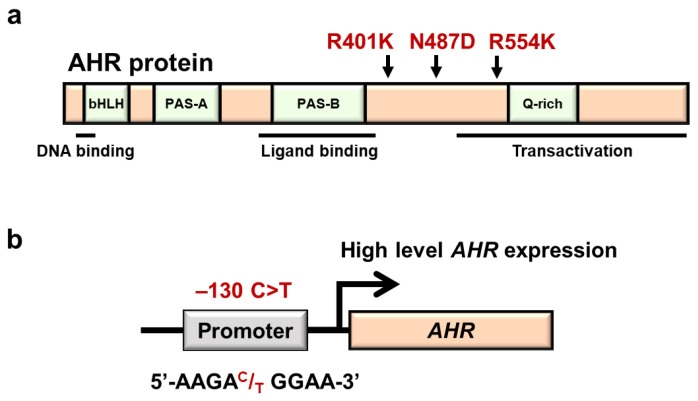
Three nonsynonymous SNPs and one regulatory SNP in the *AHR* gene. (**a**) Location of three nonsynonymous *AHR* SNPs associated with AHR domain structures. Human AhR protein contains several functional domains. bHLH, basic helix-loop-helix; PAS, P-ARNT-S; Q-rich, glutamine rich. (**b**) Location of rSNP-130 in the *AHR* gene. This rSNP has been shown to increase transcriptional regulation of the *AHR* gene.

The human *AHR* gene is located on chromosome 7p15, and many genetic polymorphisms have also been reported in the human *AHR* gene. Of the many genetic polymorphisms in the coding region of the human *AHR* gene, three nonsynonymous mutations were characterized in detail (Figure 6a). The most common SNP in this gene is the 1661 G>A (rs2066853) missense mutation, which causes an arginine to lysine substitution at codon 554 (R554K) [73], which resides in the transactivation domain of AHR. The MAF of AHR R554K varies depending on the ethnic background: 0.45 in Africans, 0.34 in Asians and 0.10 in Europeans according to the dbSNP.

It has been shown that this AHR variant is associated with a higher risk of diseases in Asia. In fact, incidences of vitiligo in Chinese [74], lung cancer among cigarette smokers in Chinese [75], coronary arterial disease in Chinese [76] and breast cancer in Thailand [77] are increased in the populations harboring these variants, suggesting that the R554K polymorphism affects the inducibility of AHR target genes and influences the onset or progression of human diseases.

However, it remains controversial whether the AHR R554K polymorphism has a positive or negative effect on AHR activity. Smart et al. reported that when induced by 3-methylcholanthrene (3-MC) in human lymphocytes, the AHR R554K variant increases the *CYP1A1* mRNA level higher than that in wild-type lymphocytes [78]. In contrast, Koyano et al. reported that the variant does not alter the *AHR* mRNA and AHR protein levels in HeLa cells [79]. Wong et al. showed that *CYP1A1* mRNA expression levels did not change substantially between murine hepatoma Hepa-1 cells transfected with human AHR R554K and wild-type AHR when induced with TCDD [80]. These results suggest that the molecular mechanism by which the R554K variant acts awaits further investigation. We speculate that the influence of R554K substitution may be context dependent.

A couple of nonsynonymous SNPs in the *AHR* gene resulting in K401R (1202 A>G, undetermined rs number) and N487D (1459 A>G, rs75519181) substitutions have also been reported with MAF 0.01 and 0.002 in the Japanese population, respectively [73,79]. Because both AHR variant proteins are easily degraded through the proteasome pathway, the expression levels of these mutant AHR proteins are reduced to approximately half of the wild-type AHR level in HeLa cells [79]. Furthermore, when the expression of the *CYP1A1*-based luciferase reporter was induced by cotransfection of AHR mutant K401R or N487D along with β-naphthoflavone (BNF)- or 3-MC-treatment, the expression levels of luciferase reporter activity were reduced to approximately half of those induced by wild-type AHR. One plausible explanation for the latter observation is that ligand binding makes these mutant AHR proteins further unstable [81]. The phenotypic consequences of both variants are not available at present.

In addition to the SNPs within the coding region of the *AHR* gene, a rSNP at −130 C>T (rs10249788) has also been reported in the *AHR* upstream promoter region (Figure 6b). The ethnicity of this rSNP has been reported [82]; the MAF of −130 C>T is 0.01 in Africans, 0.14 in Asians and 0.01 in Europeans, based on the dbSNP. This rSNP has been shown to affect transcriptional regulation of the *AHR* gene [83]. *AHR* mRNA, AHR protein and TCDD-induced *CYP1A1* mRNA levels in the minor T/T genotype are higher than those in the C/C genotype in normal human chorionic stromal cells [83]. Intriguingly, it has been reported that Chinese AD patients with both 1661G>A (R554K) and −130 C>T SNPs in combination show a higher risk of severe dry skin, suggesting that both SNPs may affect the formation of the skin barrier [84]. The influences of these SNPs are expected to be clarified in a much deeper layer.

## 9. Conclusions

Both the AHR system and the NRF2 system function as sensor-effector machineries connecting external signals to internal cellular responses. Both systems act against environmental stresses in various tissues, including the skin. Recent advances in functional dissections have revealed that the AHR and NRF2 systems are involved in the maintenance of epidermal homeostasis, in addition to the well-known roles both systems play in detoxification. In this review, we have described recent knowledge about AHR and NRF2 involvement in susceptibility to environmental stresses and the pathogenesis of AD.

Clinical phenotypes of AD are heterogeneous [85,86]. AD is also known as a multifactorial skin disease resulting from complex interactions between genetic and environmental factors. Intense pruritus is a common symptom of AD that impairs quality of life including sleep [87]. However, we do not have curative therapies for AD. Reducing skin inflammation and pruritis is a primary goal of treatment. In this regard, large-scale cohorts and biobanks have been established that enable the utilization of human specimens and integrated data from cohort studies [88]. We expect that in the near future research aiming to identify specific populations that are more susceptible to environmental stress will emerge, and through such studies, we can challenge a comprehensive understanding of the pathogenesis of AD. While the current standard treatments of AD do not focus on the individual pathogenesis mechanisms of AD, we will be able to develop targeted and personalized medicine for AD.

## Figures and Tables

**Table 2:** Effects of air pollutants on the risk of atopic dermatitis (meta-analysis).

Pollutants		Odds Ratio (95% CI)	Reference		
Tobacco smoke	Active smoking	1.87 (1.32–2.63)	Kantor et al.	2016	[55]
	Passive smoking	1.18 (1.01–1.38)			
	Maternal smoking	2.95 (2.43–3.60)	Ng et al.	2020	[56]
PM2.5		1.04 (0.96–1.12)	Ngoc et al.	2017	[51]

## Abbreviations

ABC	ATP-binding cassette
AD	atopic dermatitis
AHR	aryl hydrocarbon receptor
AHRR	aryl hydrocarbon receptor repressor
ARE	antioxidant response element
ARNT	aryl hydrocarbon receptor nuclear translocator
BNF	β-naphthoflavone
bHLH-PAS	basic helix-loop-helix/PER-ARNT-SIM
bZIP	basic leucine zipper
caNrf2	constitutively active Nrf2 mutant
CDDO-Im	1-(2-cyano-3,12,28-trioxooleana-1,9(11)-dien-28-yl)-1H-imidazole
ChIP	chromatin immunoprecipitation
CNC	cap‘n’collar
CsMBE	CNC-sMAF binding element
CYP	cytochrome P450
dbSNP	the single nucleotide polymorphism database
dnNrf2	dominant-negative form of Nrf2
EDC	epidermal differentiation complex
EGF	epidermal growth factor
EMSA	electrophoretic mobility shift assay
EpRE	electrophile response element
FLG	filaggrin
GCLC	glutamate-cysteine ligase catalytic subunit
GST	glutathione *S*-transferase
Hif1α	hypoxia-inducible factor 1α
HO-1	heme oxygenase-1
HRNR	hornerin
HSP90	heat shock protein 90
ISAAC	the international study of asthma and allergies in childhood
IVL	involucrin
jMorp	Japanese multi-omics reference panel
K5	keratin 5
K14	keratin 14
KEAP1	kelch-like ECH-associated protein 1
LCE	late cornified envelope protein
LOR	loricrin
MAF	minor allele frequency
3-MC	3-methylcholanthrene
MEF	mouse embryonic fibroblast
MRP	multidrug-resistance-associated protein
NAC	*N*-acetyl cysteine
NEO	neomycin resistance cassette
NQO1	NAD(P)H:quinone oxidoreductase 1
NRF2	nuclear factor erythroid 2-related factor 2
PCB	polychlorinated biphenyl
PM	particulate matter
RPTN	repetin
rSNP	regulatory single nucleotide polymorphism
SB	stratum basale
SC	stratum corneum
SG	stratum granulosum
Slpi	secretory leukocyte protease inhibitor
sMAF	small musculoaponeurotic fibrosarcoma
SNP	single nucleotide polymorphism
SPRR	small proline-rich protein
SS	stratum spinosum
tBHQ	*tert*-butyl hydroquinone
TCDD	2,3,7,8-tetrachlorodibenzo-*p*-dioxin
TEWL	transepidermal water loss
UDP	uridine diphosphate
UGT	uridine diphosphate -glucuronosyl transferase
XAP2	hepatitis B virus X-associated protein 2
XRE	xenobiotic response element
